# Emotional overload in Bulimia Nervosa: an ERP study of emotion processing and regulation

**DOI:** 10.1186/s40337-025-01245-7

**Published:** 2025-05-01

**Authors:** L. Vuillier, Z. Wang, S. Hassan, A. Harrison, M. P. Somerville, X. He

**Affiliations:** 1 Department of Psychology, Bournemouth University, Poole, UK; 2 Department of Psychology, Durham University, Durham, UK; 3 UCL Institute of Education, University College London, London, UK

**Keywords:** Bulimia Nervosa, Eating disorders, Emotion, Emotion processing, Emotion regulation, Neuroimaging, LPP

## Abstract

**Objective:**

People with Bulimia Nervosa (BN) self-report difficulties processing and regulating emotions. However, self-reports have been shown to be biased, particularly with people with BN who have difficulties describing their emotions. Self-reports also cannot easily disentangle between early *processing* and later *regulatory* stages, so it is not clear whether people with BN really do process their emotions more intensely or whether this is due to the aftermath of regulatory difficulties. This study aimed to use an objective way to measure (1) whether people with BN process their emotions with higher intensity compared to healthy controls (HC) and (2) whether they can successfully implement an emotion regulation strategy called cognitive reappraisal.

**Methods:**

We developed a neuroimaging task using electroencephalography to answer these questions, using the Late Positive Potential (LPP) as an objective measure of emotional arousal at the processing and regulatory stages. We tested the task in females with BN (N = 32) and matched HC (N = 35).

**Results:**

We found that our BN group showed higher LPP compared to our HC group when viewing emotional pictures, demonstrating increased emotional intensity at the processing stage. We also found that the LPP for reappraisal took longer to get back to baseline for our BN group compared to the maintain condition and our HC group.

**Discussion:**

Our results suggest that people with BN process their emotions with higher intensity and may struggle to implement subsequent cognitive reappraisal strategies when affect is high. This has direct implications for clinicians who should be aware that when evoking affect in treatment, people with BN may need greater support in understanding and managing their emotions. Clinicians may also want to offer distress tolerance skills to reduce emotional arousal before suggesting using cognitive reappraisal skills to manage strong emotions.

## Abstract (plain language summary)

Research has shown that people with Bulimia Nervosa (BN) often have trouble managing their emotions. We wanted to understand why this happens. For example, we were wondering whether people with BN feel emotions more strongly than others, or do they just have trouble finding ways to cope with their feelings? Or maybe both? We created a new task to look at what the brain is doing when people with BN experience and manage their emotions. We tested the task in 32 females with BN and 35 healthy females with no eating disorders. The results showed two main things. First, we showed that women with BN tend to feel emotions more intensely than those without BN. Second, we found that women with BN may have difficulties using cognitive reappraisal, a strategy that involves looking at situations from different angles to find more positive aspects. These findings are important because they suggest that people with BN might need extra help in two areas: they may need support in dealing with the strength of their emotions, and in finding alternative ways to manage their feelings and reduce distress. This research could lead to better ways to support people with BN in the future.

Eating disorders (EDs) are serious mental health conditions that have among the highest morbidity and mortality rates of all psychiatric illnesses, with suicide a major cause of death [1, 2]. Bulimia nervosa (BN) is one of the most common EDs and is characterised by bingeing episodes followed by inappropriate compensatory behaviours such as excessive exercise, purging, fasting, and laxatives [3]. There is a growing body of evidence that shows an association between emotions and EDs, particularly with binge-eating and purging which are thought to be used as an attempt to lower negative affect in BN [4, 5]. However, most research looking at emotions in EDs relies on questionnaires, which is problematic because questionnaires are often not able to disentangle *frequency* of use vs success in *implementing* a strategy, or difficulties in *processing* vs *regulating* emotions [6, 7]. Moreover, people with BNs are known to have high levels of alexithymia [8] characterised by difficulties identifying and describing emotions, making the use of self-reports questionable. Currently, over 60% of patients with BN do not obtain complete abstinence from core ED symptoms after treatment [9], in part because current evidence-based treatments principally focus on the symptoms (e.g. the bingeing and purging episodes), rather than what is driving them, such as difficulties processing and regulating emotions [10–12]. This study uses electroencephalograpy (EEG, a technique of measuring electric brain activities) as an objective approach to explore the underlying neural mechanisms behind the self-reported difficulties with emotion processing and regulation in BN. This is important because it could help better understand the emotional difficulties faced by people with BN, and develop innovative treatments that address these. 

There is ample literature showing that people with BN have difficulties processing and regulating emotions [13]. The literature has also shown that people with BN tend to under-use commonly called ‘adaptive’ strategies, such as cognitive reappraisal [14, 15], which has been linked to many positive physical and mental health outcomes [16, 17] such as increased resilience in periods of stress [17]. The reason behind this under-use of cognitive reappraisal in people with EDs is, however, currently unknown. It is generally assumed that people with EDs are not using this strategy because they do not select it from their emotion regulation repertoire, preferring instead to rely on other strategies instead such as rumination, suppression, or even ED behaviours such as bingeing and purging [13]. However, it remains a possibility that they do not use this strategy because they cannot successfully *implement* it (i.e. it does not work to reduce their distress). Much of the existing research examining emotion regulation strategy relies on self-report methods, an approach which does not differentiate between frequency—how often someone uses a particular strategy (also referred to as habitual emotion regulation, patterns of emotion regulation, or emotion regulation tendency), and success of implementation—whether it works to reduce distress when used (also referred to as emotion regulation effectiveness, ability, or capacity; [16, 18]. This is important because cognitive reappraisal features prominently in Cognitive Behavioural Therapy (CBT), the current psychological gold standard treatment for BN [19]. As such, testing the possibility that cognitive reappraisal may not work to reduce distress in people with BN could help develop alternative treatment options.

People with BN have also been shown to experience emotions with high intensity and have elevated levels of emotional reactivity [20, 21]. However, whilst perceptual and regulatory processes are theoretically independent [6], they are in practice difficult to disentangle using questionnaires. Indeed, the process model of emotion regulation suggests that early perceptual difficulties such as increased intensity can *cause* difficulties at the regulation stage (e.g. see [6] for further detail on the process model), but the literature also suggests that emotion dysregulation may *lead to* increased emotional intensity [20, 22, 23]. Specifically, the literature is not yet clear whether the self-reported emotional intensity arises from deficits at the regulation stage, or whether people with BN suffer from a double emotional burden of increased emotional intensity at the processing stage, as well as difficulties at the regulating stage.

Electroencephalography (EEG), with the use of Event-Related Potentials (ERPs) opens a new avenue to explore and disentangle perceptual and regulatory processes. For example, the Late Positive Potential (LPP), a positive-going component maximal at parietal-occipital sites (e.g., POz), reflects the amount of processing resources, or emotional arousal, a person allocates to a stimulus [24–26] making it a great tool to evaluate emotion processing. As such, viewing emotional images evokes stronger LPP than neutral ones [25, 27]. The LPP can also be modulated by emotion regulation instructions such as distraction or suppression [28, 29], or cognitive reappraisal [30]. For example, re-evaluating an image in a more positive light (i.e. using cognitive reappraisal) leads to a diminished LPP in adults with and without psychopathology [25, 30, 31]. This makes the LPP an excellent neurophysiological marker to measure emotion processing and regulation.

Whilst no research so far has specifically investigated the processing and regulation of emotional responses in EDs, some research has used the LPP in response to food stimuli. Sarlo et al. [32] found that healthy women with bulimic tendencies showed enhanced LPP amplitudes when asked to reappraise pictures of high-caloric food, compared to women without these tendencies, suggesting they found it harder to reappraise pictures of food as less tempting. Another study [33] found that in people with Binge Eating Disorder, pictures of food created more emotional arousal (i.e. higher emotional reactivity), as depicted by higher LPP, compared to healthy controls, although they did not look at reappraisal processes. It is however important to note that looking at the perception and reappraisal of food items using LPP may not generalise to emotional scenes. One study did look at emotion processing and down-regulation of emotional pictures in people with anorexia nervosa (AN) [34] and found no significant differences in LPP, both in terms of emotion processing and emotion regulation in their AN vs healthy control group. However, their instruction for down-regulating emotions was to “*reduce the emotional response that it might elicit*” so it is not clear what strategy their participants used, which may not have been reappraisal. Also, whilst difficulties with emotion regulation are thought to be transdiagnostic [13], differences between ED categories can be observed, such that dysfunctional emotion regulation is associated with different outcomes in AN vs BN [35] and there may also be differences in the use of adaptive strategies such as reappraisal between AN and BN [36].

This study aimed to explore the underlying mechanisms behind the self-reported emotional intensity and low usage of cognitive reappraisal in people with BN. Specifically, it aimed to determine whether females with BN experience emotions with high intensity, and whether they can successfully implement cognitive reappraisal (i.e., does it work to reduce distress). We developed a task based on Foti and Hajcak’s [31] which allowed us to separately study the neural bases of emotion processing and regulation and tested it in females with BN (N = 32) and matched healthy controls (HC, N = 35). We first hypothesised (H1) that our females with BN would self-report using less reappraisal than our healthy controls (as per [15], for example) and self-report experiencing their emotions with more intensity (as per Svaldi et al. [21] for example). Given the lack of clarity in the literature, we were unsure what to expect regarding the LPP when processing (H2) and reappraising (H3) negative emotions, therefore, these hypotheses were exploratory.

## Methods

### Participants

An a priori power analysis conducted using G*Power version 3.1.9.7 [37] revealed that a minimum sample size of 27 participants per group would be sufficient to detect small to medium effect sizes (partial η^2^ = 0.03) in the interaction of a 2 × 3 factorial mixed-measures analysis of variance (ANOVA) with an assumed α = 0.05 and power of 0.80. As such we aimed to recruit 30–35 participants in each group to account for excessive movements and artifact rejection.

The study sample consisted of N = 35 female healthy controls (HC) recruited via media advertisements and university campus outreach between August 2019 and August 2023. They took part in a pre-selection survey and only those scoring below two on the EDE-Q and reporting no ED behaviour and no current mental health diagnosis were included. We also recruited N = 32 females with symptoms of Bulimia Nervosa (BN). Out of these, N = 4 were recruited from an ED service. The rest (N = 28) were recruited via media advertisements and university campus outreach. Although not all had an official diagnosis of BN^1^, all met the Diagnostic and Statistical Manual of Mental Disorders, Fifth Edition (DSM-5) criteria for a diagnosis of BN. As such, interested participants took a pre-section survey and only those scoring above 4 on the EDE-Q total score and reporting at least four episodes of bingeing and four episodes of inappropriate compensatory behaviour over the past 28 days on the EDE-Q were invited to take part. Whilst participants from the control group were excluded if they had any current mental health diagnosis, participants in the BN group reported a range of comorbid diagnoses, including depression (N = 10), anxiety (N = 10), OCD (N = 1) and PTSD (N = 2). Groups were matched in terms of age, ethnicity, and education level. See Table 1 for the demographic descriptions of the sample.

**Table 1:** Sample description

	BN group (N = 32)	HC group (N = 35)	Test of group differences
EDE-Q: total	4.5 (0.76; 2.9–5.9)	0.6 (0.43; 0–1.8)	t(65) = −26.5, *p* < 0.001
EDE-Q: binge	11.8 (7.8; 0–28)	0.2 (0.87; 0–5)	X^2^ (16, N = 66) = 59.4, *p* < 0.001
EDE-Q: compensatory behaviours	15.7 (16.6; 0–68)	0.9 (2.8; 0–15)	X^2^ (22, N = 67) = 47.2, *p* < 0.001
Age	23.4 (6.8; 18–29)	24.4 (5.6; 18–23)	t(63) = 0.63, *p* = 0.529
Ethnicity	Asian (n = 3) Black (n = 1) Mixed (n = 1) White (n = 27) Other (n = 0)	Asian (n = 5) Black (n = 1) Mixed (n = 0) White (n = 25) Other (n = 2)	X^2^ (4, N = 67) = 3.7, *p* = 0.444
Education level	College (n = 3) Further education (n = 16) Undergraduate level (n = 10) Postgraduate level (n = 3)	College (n = 3) Further education (n = 13) Undergraduate level (n = 14) Postgraduate level (n = 5)	X^2^ (3, N = 67) = 1.35, *p* = 0.718

Numbers represent mean, with SD followed by min–max in brackets, unless specified

Data analysis excluded two healthy participants and three participants with BN due to excessive muscular movements (n = 4), or technical issues (n = 1). This study obtained ethical approval from the Science, Technology & Health Research Ethics Panel at Bournemouth University (ID:26501) as well as from the National Health Service (NHS) (IRAS ID: 254746). All participants provided informed consent and were debriefed post-study.

### Questionnaires

#### Eating disorder examination questionnaire (EDE-Q)

The EDE-Q [38] contains 28 questions referring to the past 28 days, such that high scores indicate severe ED psychopathology. Questions are scored from 0 to 6 with a maximum possible total average score of 6. A score equal to or above 4 is commonly used to classify individuals within the clinical range (e.g. [39]), with a score of 2 or below considered representative of a community sample without ED behaviours [40]. The EDE-Q also contains six questions measuring: binge eating (i.e. eating an unusually large amount of food with a sense of having lost control over the eating); purging; laxative uses; and excessive exercise as a means of controlling their shape or weight. The EDE-Q total score has good internal consistency which was confirmed in our sample (BN α = 0.84; HC α = 0.88).

#### Emotional regulation questionnaire (ERQ)

The ERQ [16] is a 10-item scale designed to measure respondents’ tendency to regulate their emotions using Cognitive Reappraisal or Expressive Suppression. The six questions to measure cognitive reappraisal are scored on a 7-point Likert-type scale, with a maximum score of 42 (a higher score means more tendency to use this strategy). The ERQ has good internal consistency which was also confirmed in our sample (BN α = 0.87; HC α = 0.92 for cognitive reappraisal).

#### Emotional reactivity scale (ERS)

The ERS [20] is a 21-item scale that measures emotional sensitivity, intensity and persistence. We only used the seven questions to measure intensity (Items 3, 4, 6, 17, 19, 20, 21). Questions are scored on a 5-point Likert-type scale (0–4), with a maximum score of 28 for intensity (higher score means more intensity). The ERS has good internal consistency which was confirmed in our sample (BN α = 0.90; HC α = 0.82).

### The emotion regulation task

**Fig. 1:**
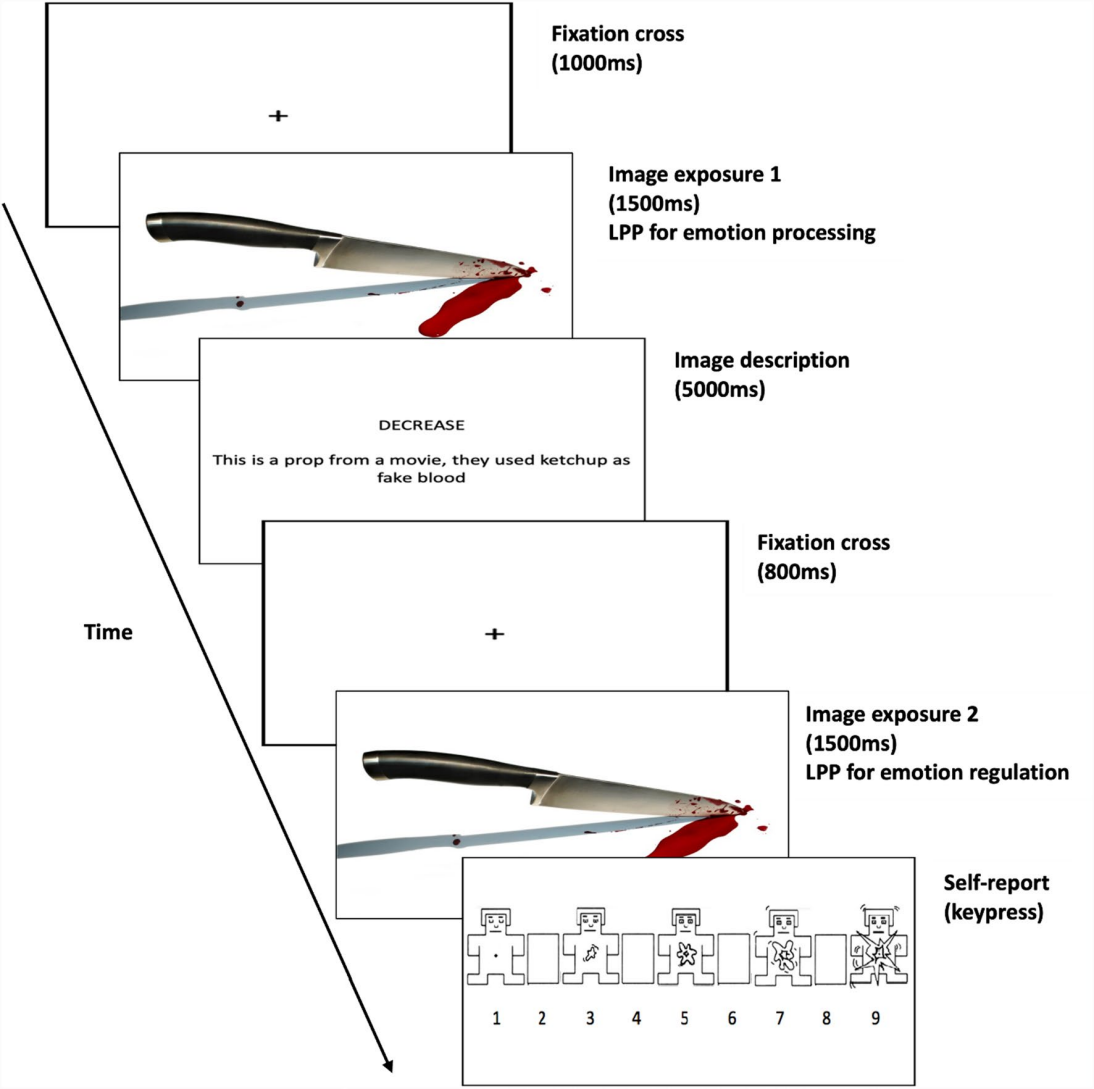
Example of a reappraisal trial sequence in the emotion regulation task. Due to copyright rules, the real IAPS images are not used in this figure. The maintain, decrease and view conditions all followed the same format, except with different instructions. The self-report Likert scale included pictorial icons from the Self-Assessment Manikin Scale [42]

Participants were seated in a dimly illuminated testing booth. The visual stimuli were displayed in the centre of a 17-inch PC monitor at a viewing distance of 60 cm. The emotion regulation task had three conditions: Neutral, Maintain, and Reappraisal. Each of these conditions featured 46 unique pictures from the International Affective Picture System (IAPS; [41]), so that each picture was only presented once. Pictures and conditions were randomised across three blocks of 46 trials each, so that each block contained neutral as well as negative pictures. Each block contained two-thirds of negative pictures (one third for reappraisal and one third for maintain trials) and one-third of neutral pictures, for a total of 46 pictures per block. In total, there were 92 negative images (46 for reappraisal and 46 for maintain) and 46 neutral images. There were two versions of the task which were counterbalanced using different IAPS images so that each participant only saw each picture once (with two exposures per pictures). As per Fig. 1, a trial began with a 1000 ms fixation display, succeeded by a neutral or negative picture shown for 1500 ms. Following the picture, participants encountered a text prompt for 5000 ms, with the content varying based on the (randomised) condition. For the neutral condition (only for neutral pictures), the instruction asked participants to observe the image without any specific emotional engagement. For the maintain condition (for negative pictures), the instruction encouraged participants to fully engage with their emotional responses (e.g. *“The ambulance crew arrived too late and could not save the driver”* following the picture of a car accident). For the reappraisal condition (for negative pictures), the instructions aimed to guide participants to reduce their response to the picture by cognitively reframing the negative image, either through focusing on a more positive outcome of the emotional scene (e.g. *“All people were saved thanks to the ambulance crew's hard work”* following the picture of a car accident), or through objectifying the situation by viewing it as fake, from a movie for example. They were told to specifically reframe their interpretation using the description and to not think of something else^2^. See Supplementary Material for the full list of the IAPS pictures and corresponding descriptions. After the text prompt, there was a subsequent 800 ms fixation period, followed by the re-presentation of the identical image for another 1500 ms. Following this second image display, participants were required to assess and report the strength of their emotional response (i.e. arousal), utilising the Self-Assessment Manikin scale [42] for arousal.

### EEG recording and data analysis

*EEG recording*. Continuous EEG data were captured using an active electrode system (BrainAmp DC, Brain Products, GmbH, Gilching, Germany) from 32 scalp electrodes. These electrodes were positioned according to the extended 10–20 system [43] at designated sites including AFz, Fz, FCz, Cz, CPz, Pz, POz, F3, F4, F7, F8, FC3, FC4, FT7, FT8, CP3, CP4, CP5, CP6, P3, P4, P7, P8, PO3, PO4, PO7, PO8, O1, and O2. Additionally, two electrodes were placed on the mastoid bones behind each ear. The vertical electrooculogram (VEOG) was recorded from electrodes below the left eye, and the horizontal electrooculogram (HEOG) from electrodes adjacent to the outer canthi of both eyes.

EEG data were recorded using BrainVision Recorder (BrainVision Recorder, Version 1.23.0001, Brain Products GmbH, Gilching, Germany) at a sampling rate of 1000 Hz. The raw EEG data were band-pass filtered from 0.01 to 30 Hz. All channels were referenced online to the left mastoid electrode and re-referenced offline to an average of both left and right mastoids. Independent component analysis (ICA) was performed on the continuous data to identify and remove eyeblink and eye movement [44]. Artifact rejection was performed for individual channel with trials contaminated with muscular movement artefacts (exceeding ± 80 µV), abnormally rapid signal change (over 50 μV/ms gradient), and low activities (consistently lower than 0.5 µV throughout any 200 ms duration) using all other channels being removed as artefacts from EEG analysis. To analyse the ERP components, the remaining EEG was segmented into 1600 ms epochs ranging from 100 ms before to 1500 ms after the onset of the image display to capture the full image presentation. The 100 ms pre-stimulus interval was used for baseline correction. To calculate LPPs for each condition, EEG was averaged separately for each group (BN or HC) and for each condition of emotion processing (from 100 ms before to 1500 ms after the onset of the first exposure to the images; Image types: negative and neutral) and emotion regulation (from 100 ms before to 1500 ms after the onset of the second exposure to the image after the instructions; Instruction types: reappraisal, maintain, and neutral). All EEG data processing was performed with BrainVision Analyzer software (Brain Products GmbH, Gilching, Germany). 

*ERP and statistical analysis.* Based on a collapsed localisers approach [45], LPP was quantified as the average activity where it was maximal on the scalp at POz electrode. Statistical evaluations were performed using JASP statistical software (version 0.18.1.0, www.jasp-stats.org). One-way (Group: BN vs HC) between-subject Analyses of Variance (ANOVA) was conducted for H1. A 2 (Group: BN vs HC) × 2 (Image type: Negative vs Neutral) mixed-design ANOVA supplemented by permutation-based false discovery rate (FDR) estimates [46] was conducted for H2. Finally, a 2 (Group: BN vs HC) × 3 (Instruction type: Reappraisal, Maintain vs Neutral) mixed-design ANOVA supplemented by permutation-based FDR estimates for H3. Permutation-based FDR estimates were performed using RStudio (Version 2022-02-03-492, RStudio, 2022, based on the R programming language Version 4.2.0, R Core Team, 2022).

## Results

### **Hypothesis 1**

Self-report use of cognitive reappraisal and emotional reactivity

As predicted, we found that the BN group (*M* = 25.0, *SD* = 8.1) reported less use of reappraisal on the ERQ compared to the HC group (*M* = 29.5, *SD* = 7.4; *F*(1,60) = 5.1, *p* = 0.028, ${\eta }_{p}^{2}$ = 0.08). We also found that the BN group self-reported experiencing their emotions with higher intensity (*M* = 17.7, *SD* = 6.8) compared to the HC group (*M* = 10.8, *SD* = 5.0; *F*(1,60) = 20.7, *p* < 0.001, ${\eta }_{p}^{2}$ = 0.26).

### **Hypothesis 2**

LPP for emotion processing

**Fig. 2:**
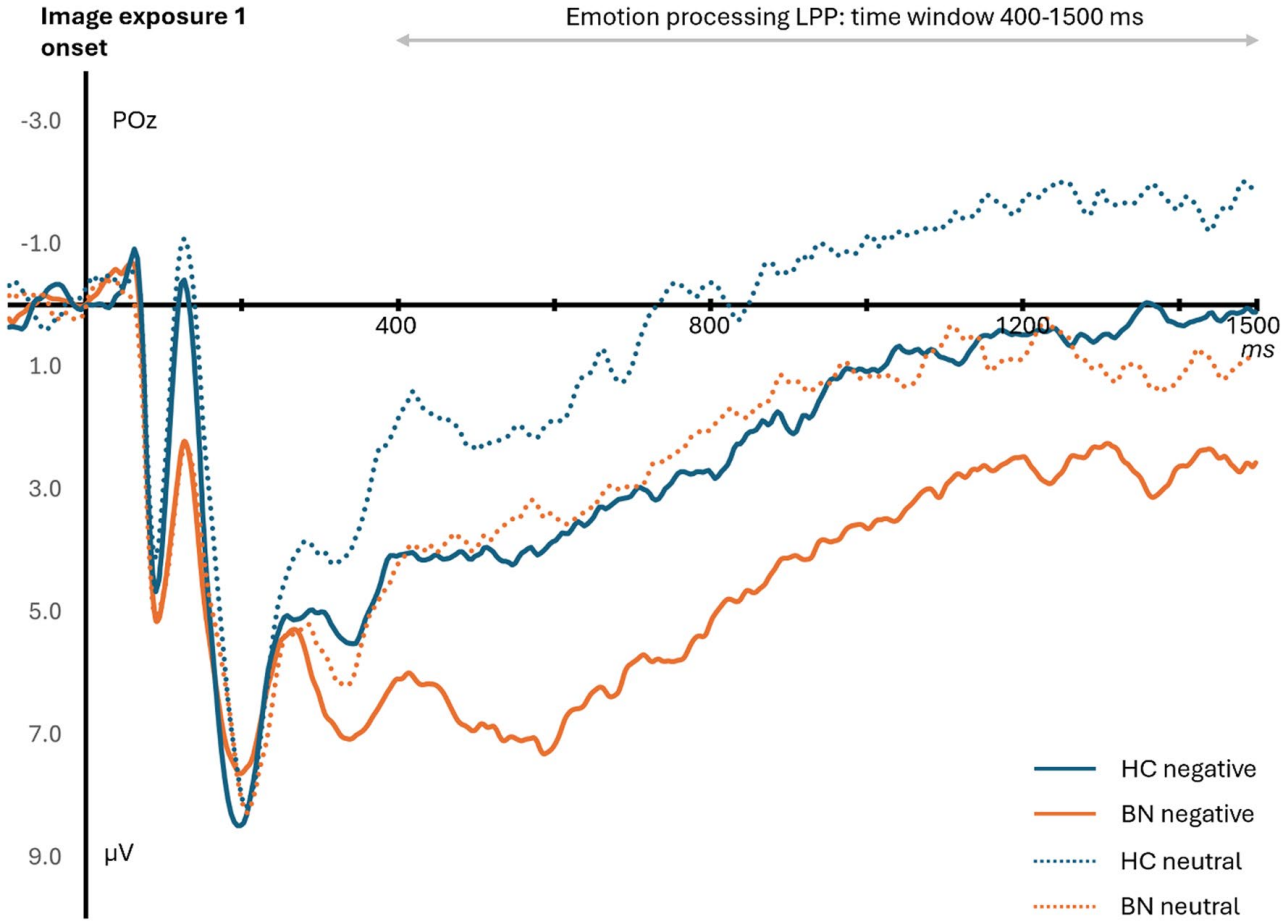
Grand-averaged LPPs for emotion processing with the healthy control group (HC) in blue and the Bulimia Nervosa group (BN) in orange. ERPs were triggered at the electrode site POz for images presented before instructions (exposure 1). Images were randomly selected between negative (straight lines) and neutral (dot lines) conditions. The LPP time windows were 400–1500 ms after the onset of each image (here shown at 0 ms)

Differences in emotion processing were assessed using a 2 (Group: BN vs HC) × 2 (Image type: Negative vs Neutral) mixed-design ANOVA to examine whether the BN group experienced higher arousal level than the HC group when negative or even neutral images were presented. A main effect of Group revealed that LPP amplitudes were significantly larger (*F*(1, 60) = 4.74, *p* = 0.033, ${\eta }_{p}^{2}$= 0.07) in the BN group (*M* = 3.0, *SD* = 4.1), compared to those in HC group (*M* = 0.7, *SD* = 4.4), which can be seen in Fig. 2. There was also a significant main effect of Image type, *F*(1, 60) = 57.4, *p* < 0.001, ${\eta }_{p}^{2}$ = 0.49, suggesting that for both HC and BN group, negative images (*M* = 3.0, *SD* = 4.6) triggered significantly larger LPP than the neutral images (*M* = 0.7, *SD* = 4.4). The interaction between Group and Image type did not reach significance, with *F*(1, 60) = 0.2, *p* = *0.672*, ${\eta }_{p}^{2}$ < 0.01.

The results were further supported by permutation-based t-tests with FDR correction. The negative images’ LPP components of the HC group fell back to baseline at around 880 ms, while the BN group remained significantly larger than the baseline through the whole time period (i.e. until at least 1500 ms). The BN group also showed a delayed return to baseline LPP (816 ms) in the neutral condition compared to the HC group, whose LPP returned to baseline at 372 ms.

### **Hypothesis 3**

LPP for emotion regulation

Self-rating: The self-rating arousal scores were subjected to a mixed-design ANOVA with the within-factor Instruction type (Reappraisal, Maintain vs Neutral) and between-factor Group (BN vs HC). A significant main effect of instruction type, *F*(2, 120) = 414.2, *p* < 0.001, ${\eta }_{p}^{2}$ = 0.87, revealed that the self-rating scores on the three instruction types were significantly different, with the highest rating in the Maintain condition (*M* = 6.0, *SD* = 1.7), then Reappraisal (*M* = 3.8, *SD* = 1.3), and Neutral (*M* = 1.9, *SD* = 0.7; *p* < 0.001 of post-hoc t-tests with Bonferroni correction). The BN group (*M* = 4.2, *SD* = 1.1) showed higher arousal self-ratings than the HC group, regardless of the condition, compared to the HC group (*M* = 3.6, *SD* = 1.1, *F*(1, 60) = 4.2, *p* = 0.046, ${\eta }_{p}^{2}$ = 0.07). The interaction term of Instruction type × Group was marginal, *F*(2, 120) = 3.3, *p* = 0.054, ${\eta }_{p}^{2}$ = 0.05.

**Fig. 3:**
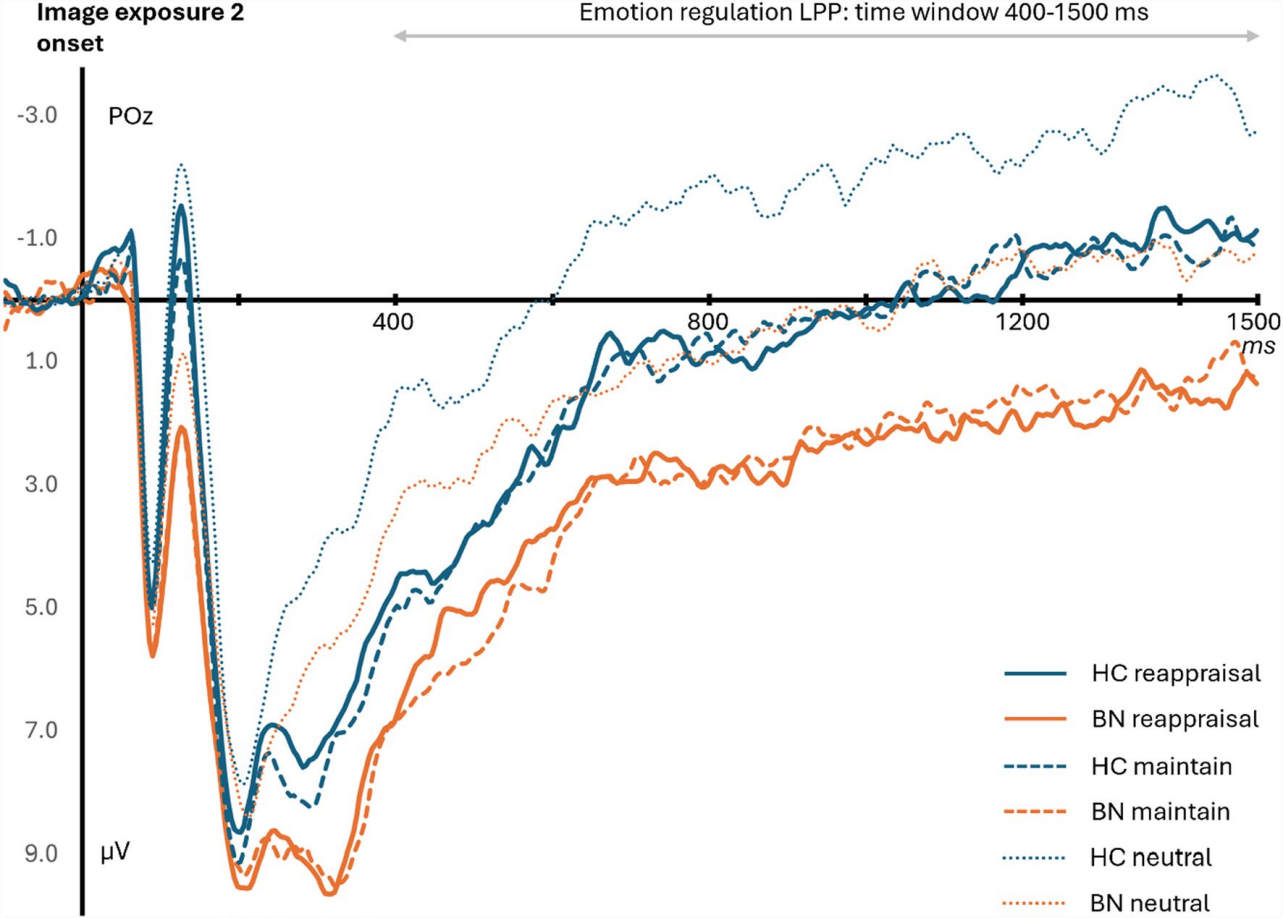
Grand-averaged LPPs for emotion regulation with the healthy control group (HC) in blue and the Bulimia Nervosa group (BN) in orange. ERPs were triggered at the electrode site POz for negative images presented after instructions (exposure 2), which were randomly selected between reappraisal (straight lines), maintain (dash lines), and neutral (dotted lines) conditions. The LPP time windows were 400–1500 ms after the onset of each image (here shown at 0 ms)

*LPPs*: The same ANOVA on LPP mean amplitudes only revealed significant main effects of Instruction type (*F*(2, 120) = 25.7, *p* < 0.001, ${\eta }_{p}^{2}$ = 0.30) and Group (*F*(1, 60) = 7.1, *p* = 0.010, ${\eta }_{p}^{2}$ = 0.11), as seen in Fig. 3. The BN group (*M* = 2.0, *SD* = 3.5) showed higher LPP amplitudes compared to the HC group (*M* = -0.1, *SD* = 2.6), but there was no condition interaction. Further post-hoc t-tests suggested that, overall, the Neutral LPP (*M* = -0.6, *SD* = 3.6) was significantly smaller than the LPP in the Reappraisal (*t*(61) = 6.2, *p*_*bonf*_ < 0.001) and Maintain conditions (*t*(61) = 6.2, *p*_*bonf*_ < 0.001).

Estimates by permutation-based one-sample t-tests with FDR correction further identified that the BN group’s LPP component for the reappraisal condition remained significantly different from baseline until 1344 ms, whereas the HC group’s LPP component returned to baseline from 644 ms. This demonstrates that the BN group took longer than the HC group to reappraise their emotions, and they still showed high arousal until right before the picture disappeared. The same permutation-based analyses for the maintain condition showed that the BN group’s LPP returned to baseline more quickly than in the reappraisal condition, as the cluster became insignificant from 924 ms, suggesting that using reappraisal led to more sustained arousal than simply maintaining the emotional impact of the negative pictures in the BN group. The HC group’s LPP in the maintain condition returned to baseline at 636 ms, which is around the same time as for the reappraisal condition. In the Neutral condition, both groups’ LPP returned to baseline around the 500 ms mark (532 ms for the BN group; and 516 ms for the HC group).

## Discussion

This study aimed to explore the neural bases of emotion processing and regulation in people with Bulimia Nervosa. We developed an EEG task which we tested in females with BN (N = 32) and matched HC (N = 35). We first confirmed that our BN group self-reported experiencing their emotions with higher intensity and self-reported lower use of reappraisal compared to our HC group, confirming H1. We also found that our BN group showed higher LPP compared to our HC group when viewing emotional pictures (H2). Our results also showed that participants with BN showed large LPP in the reappraisal condition, whilst the LPP returned to baseline earlier on for our control participants, suggesting difficulties implementing cognitive reappraisal in our BN participants (H3). We discuss these results in turn.

It was no surprise to find that our participants self-reported more emotional intensity and low usage of cognitive reappraisal compared to our HC as this has been reported in the literature [13, 21]. However, it was interesting to see that the self-reported emotional reactivity was reflected in the more objective measure using the LPP. The literature is unclear as to whether emotional reactivity and emotion regulation are one or two processes, as some authors suggest that emotional reactivity may *cause* difficulties regulating emotions while others suggest that emotion dysregulation may *lead to* emotional reactivity, while yet others suggest that these concepts are actually indistinguishable [20, 22, 23, 47]. Our experimental paradigm allowed us to separate these processes by looking at emotional arousal during the processing stage, independently of the regulatory stage, and we found that people with BN do seem to process their emotions with higher intensity, and that this is not a byproduct of poor emotion regulation skills. This is important because it suggests that new interventions working on emotions for people with BN should target emotional intensity. For example, training could help them identify and label their emotions—which is something this group finds challenging [8]—because alexithymia is associated with emotional reactivity [48]. A recent study showed that helping people with EDs identify and label their emotions (amongst others) was linked to reduced alexithymia and eating disordered behaviours [49], and whilst the authors did not measure whether this improvement was mediated by reduced emotional reactivity, it is a possibility that future research should explore.

Our study also reported on difficulties successfully *implementing* cognitive reappraisal, which had not yet been explored in people with BN. Although we were surprised to not find a group × instruction interaction for H3, we suggest that this is due to using long time widows for the LPP (1100 ms). Some researchers have in the past split long LPPs into early, mid and late LPP windows, but this simplicity and variability of defining LPP time windows has been criticised [50], and Permutation-based FDR estimates have been recommended instead [46].. Our permutation-based FDR estimates did show a later return to baseline for our BN group in the reappraisal condition, compared to the maintain condition or the HC group, suggesting difficulties implementing reappraisal. Whilst this is the first study demonstrating difficulties implementing cognitive reappraisal in BN, this was not completely surprising. For example, it is a well-known finding that people tend to refrain from using cognitive reappraisal in high-intensity emotion situations, for example preferring to use distraction instead [51–55]. Cognitive reappraisal relies on cognitive resources in working memory [56], so when cognitive resources are already recruited to *attend to* an emotional event, there may not be enough left to *regulate* the emotion. This suggests that offering people distress tolerance skills, for example distraction techniques to disengage from the emotional event at an early attentional stage, may be more helpful. And indeed, Shafir et al. [53] showed that when healthy participants exhibited enhanced emotional intensity (measured by enhanced LPP) they were more likely to choose distraction over reappraisal, compared to low-intensity trials. Distraction has an ambiguous status in the emotion regulation literature because it essentially does not help deal with the emotion; it simply distracts the person whilst the emotion passes, but next time a similar emotional event comes back, the person will not be better equipped to deal with it [57]. However, because distraction is helpful when the cognitive load is too intense, it could potentially be suggested as an alternative to the urge to binge-eat or purge. A recently developed DBT-informed psycho-educational intervention on emotion regulation seems to support the use of distraction as an effective strategy for distress tolerance, to help “ride the intense emotional wave”, before returning to the emotional situation and use other strategies such as reappraisal once people are less emotionally aroused [49]. The current paper did not look at the effect of distraction or other distress tolerance technique on the LPP, but future research should explore whether in people with BN, distraction may reduce the LPP more than reappraisal.

The extended process model defines emotion regulation as the dynamic ways in which people detect emotions, determine that they want to regulate them, and attempt to do so through selecting and implementing a strategy, forming an iterative cycle constantly in need of updating. For example, if the existing selection and implementation decisions result in the desired outcome (for example reduced negative affect), then the person can continue relying on these decisions in the future. But if the emotion does not change (or changes in undesirable ways), then the person may decide to select a different strategy in the future [58]. Here, we showed that reappraisal does not seem to reduce distress in people with BN, which may explain why they do not select this strategy in their daily life. Cognitive reappraisal is part of cognitive-behavioural approaches that are frequently employed to challenge thoughts and feelings in Cognitive Behavioural Therapy (CBT), the current psychological gold standard treatment for BN [19]. Our findings show that simply telling people with BN to change the way they think about an emotional situation will not actually result in lower distress, particularly when experiencing emotions with high intensity. Importantly, our findings do not suggest that people with BN can never learn to use reappraisal to reduce distress. Instead, they suggest that without addressing emotional intensity first, reappraisal does not seem to be the best strategy to reduce distress in BN. We recommend that future research explore training people with BN to use distress tolerance skills, and test whether this helps them successfully reduce emotional arousal as well as implement cognitive reappraisal. Such research will be important to better understand whether the observed difficulties with reappraisal are due to heighten emotional intensity, or other factors.

Our study has many strengths, starting with the fact we used an objective measure of emotion intensity and regulation with ERPs instead of relying on self-reports which can be biased. However, our study has some limitations. First, whilst we did provide our participants with alternative explanations in the reappraisal condition (rather than asking them to come up with it themselves), they may not have followed the instructions. It was for example interesting to note that our HC group showed reduced LPP in the maintain condition, which was unexpected. Therefore, it is possible that they reduced their arousal through automatic emotion regulation strategies in the maintain condition, rather than follow our instructions to ‘maintain’ their emotional arousal. It is not clear whether asking participants to report subjectively on their ability to follow instructions after the task would have given us any insight, particularly if some emotion regulation strategies were automatically and subconsciously activated, but we recommend that future research add a post-test debrief to test this possibility. Another important limitation is that we only tested female participants for convenience purposes, despite the fact we know males with EDs also have difficulties identifying and regulating their emotions [15]. There does also seem to be sex differences in the association between reappraisal and EDs, such that low reappraisal use seems associated with higher ED cognition in females but not in males [15]. It therefore remains to be tested whether males with EDs could implement reappraisal successfully. We also only tested females with BN, and predominantly females with BN from the community, and whilst difficulties with emotion regulation are thought to be transdiagnostic [13], differences between ED categories have been observed [35, 36]. For example, Danner et al. [59] showed that individuals with binge-eating behaviours (like BN) reported using reappraisal less than women with the restrictive subtype of anorexia nervosa. Future work could expand on this understanding by involving more diverse experiences of EDs, such as those with anorexia nervosa. It is also important to note that whilst we combined people with BN as one group to compare against a ‘healthy’ group, EDs are heterogeneous. A recent paper looking at an emotion-based intervention [49] for example found some variation in people’s preferences for reappraisal, with one participant saying they found reappraisal “*particularly useful […] it helped calm me down and let me see things in perspective*”, whilst another said they have “*always struggled with reappraisal because my mind feels like it gets tied in knots and jams up with questioning everything, and doesn’t know where to stop, and I end up not having a clue how I feel*”. Incidentally the first participant was male and the second female so it could be indeed due to sex differences although this would need to be examined in more detail in future research.

## Conclusion

Our paper showed that females with BN experience their emotions with more intensity and are not as successful in using cognitive reappraisal to reduce emotional arousal compared to a healthy control group. These findings have implications for adapting current evidence-based treatment, and developing new emotion-based treatments for BN.

## Data Availability

The datasets generated and/or analysed during the current study are available in the Bournemouth Online Research Data Depository (BORDaR) repository (https://doi.org/10.18746/bmth.data.00000390) or are available from the corresponding author.

## Abbreviations

AN: Anorexia nervosa
ANOVA: Analyses of variance
BN: Bulimia Nervosa
CBT: Cognitive behaviour therapy
DSM-5: Diagnostic and Statistical Manual of Mental Disorders, Fifth Edition
ED: Eating disorder
EDE-Q: Eating disorder examination questionnaire
EEG: Electroencephalography
ERP: Event-Related Potential
ERS: Emotion reactivity scale
FDR: False discovery rate
HC: Healthy control
IAPS: International Affective Picture System
LPP: Late Positive Potential
NHS: National Health Service

## Footnotes

1 This was partly due to the COVID pandemic which saw an increase in ED service use and help seeking behaviours, creating long waiting list for assessments, and a change in the type of patients prioritised for early assessment, meaning we did not manage to recruit via ED services as planned.

2 Our paradigm differs from most other paradigms in the field on this important point. Most paradigms ask participants to come up with their own alternative explanation for reappraising the emotional picture. Whilst this may be closer to real life situations, such paradigms cannot differentiate whether a participant cannot come up with an alternative explanation or whether they do but it does not work to reduce distress. Our design, adapted from Foti and Hajcak [31] specifically provided participants with an alternative explanation to remove the untestable possibility that they may not have been able to come up with an alternative explanation.
